# Sialylation regulates neutrophil transepithelial migration, CD11b/CD18 activation, and intestinal mucosal inflammatory function

**DOI:** 10.1172/jci.insight.167151

**Published:** 2023-03-08

**Authors:** Veronica Azcutia, Matthias Kelm, Dylan Fink, Richard D. Cummings, Asma Nusrat, Charles A. Parkos, Jennifer C. Brazil

**Affiliations:** 1 Department of Pathology, University of Michigan, Ann Arbor, Michigan, USA.; 2 Department of Surgery, Beth Israel Deaconess Medical Center, Harvard Medical School, Boston, Massachusetts, USA.

**Keywords:** Immunology, Inflammation, Glycobiology, Neutrophils

## Abstract

Polymorphonuclear neutrophils (PMNs) play a critical role in clearing invading microbes and promoting tissue repair following infection/injury. However, dysregulated PMN trafficking and associated tissue damage is pathognomonic of numerous inflammatory mucosal diseases. The final step in PMN influx into mucosal lined organs (including the lungs, kidneys, skin, and gut) involves transepithelial migration (TEpM). The β2-integrin CD11b/CD18 plays an important role in mediating PMN intestinal trafficking, with recent studies highlighting that terminal fucose and GlcNAc glycans on CD11b/CD18 can be targeted to reduce TEpM. However, the role of the most abundant terminal glycan, sialic acid (Sia), in regulating PMN epithelial influx and mucosal inflammatory function is not well understood. Here we demonstrate that inhibiting sialidase-mediated removal of α2-3–linked Sia from CD11b/CD18 inhibits PMN migration across intestinal epithelium in vitro and in vivo. Sialylation was also found to regulate critical PMN inflammatory effector functions, including degranulation and superoxide release. Finally, we demonstrate that sialidase inhibition reduces bacterial peptide–mediated CD11b/CD18 activation in PMN and blocks downstream intracellular signaling mediated by spleen tyrosine kinase (Syk) and p38 MAPK. These findings suggest that sialylated glycans on CD11b/CD18 represent potentially novel targets for ameliorating PMN-mediated tissue destruction in inflammatory mucosal diseases.

## Graphical abstract

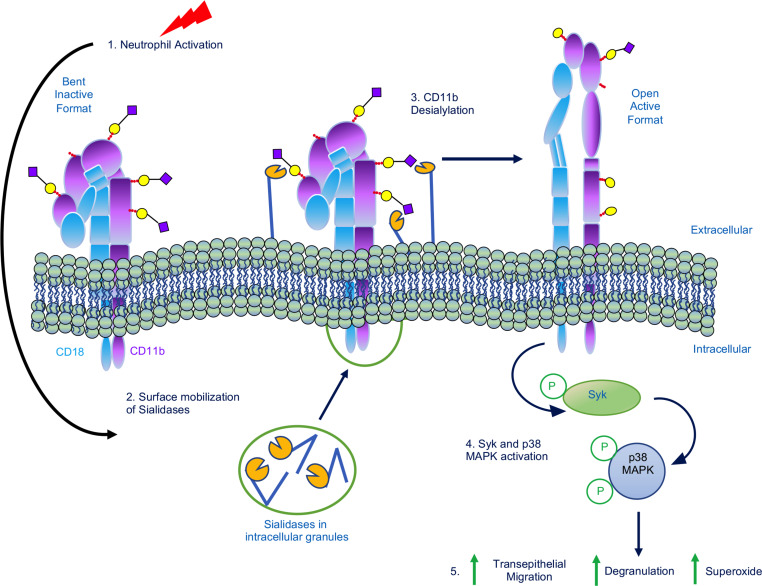

## Introduction

Polymorphonuclear neutrophils (PMNs) are the first immune responders to injury or inflammation playing critical roles in clearing invading pathogens and promoting subsequent repair and restitution of tissue homeostasis (1–3). However, dysregulated influx of PMNs coupled with indiscriminate release of proteolytic enzymes as well as reactive oxygen metabolites are pathological features of numerous diseases characterized by persistent or intermittent bursts of active inflammation. Trafficking of PMNs to inflamed tissues begins with extravasation from the microcirculation: a multistep process facilitated by a series of well-characterized ligand-receptor binding events (4–10). In the case of mucosal lined organs — including the lungs, intestine, urinary tract, kidneys, and skin — following diapedesis, activated PMN must cross a polarized epithelial barrier to arrive at sites of injury. While the molecular mechanisms that regulate PMN extravasation have been extensively characterized (9–11), much less is known about the steps that facilitate PMN transepithelial migration (TEpM). Increased understanding of PMN TEpM is of critical importance, given the direct correlation between dysregulated PMN epithelial trafficking, disease severity, and patient symptoms in many inflammatory disorders including chronic obstructive pulmonary disease (COPD), psoriasis, cystitis, arthritis, gastritis, and inflammatory bowel disease (IBD) (6, 7, 12–19).

Previous work has demonstrated a critical role for the β2 integrin Mac-1 (CD11b/CD18, αMβ2) in regulating PMN adhesive interactions with intestinal epithelium during trafficking (20–22). In addition, CD11b/CD18-mediated regulation of potentially harmful PMN effector functions, including degranulation, apoptosis, and superoxide generation, has also been reported (23–25). While precise mechanisms through which CD11b/CD18 regulates PMN function remain incompletely understood, recent glycomics studies have highlighted that terminating sugar residues at the ends of longer glycan chains on CD11b/CD18 can be targeted to regulate PMN TEpM (26, 27). While these studies suggest roles for terminal fucose and GlcNAc glycans in regulating PMN function, effects of targeting sialic acid (Sia), the most abundant terminal glycan found on PMN, were not investigated. Sia is a negatively charged monosaccharide, and as such, it plays a unique role in regulation of cell-surface charge, glycoprotein ligand affinity, and cell-to-cell adhesion interactions. Interestingly, previous studies have identified that PMN activation results in translocation of sialidases from intracellular granules to the cell surface in order to cleave Sia residues from adjacent glycoproteins, resulting in alteration of surface sialylation profiles (28, 29). Despite these observations, the role of sialylation in regulating PMN function during active mucosal inflammation has not been explored.

In this study, we demonstrate that cleavage of Sia from CD11b/CD18 has potent effects on PMN functional responses. Specifically, we show that sialidase-mediated removal of α2-3–linked Sia from PMN CD11b/CD18 is required for PMN intestinal trafficking in vitro and in vivo. Furthermore, inhibition of sialidase activity results in reduced human and murine PMN degranulation and superoxide release — key effector functions implicated in PMN-mediated mucosal tissue damage. We show that sialidase-dependent effects are secondary to reduced conformational activation of PMN CD11b/CD18, resulting in decreased spleen tyrosine kinase (Syk) signaling. These findings suggest that sialylation of CD11b/CD18 may represent a novel target for reducing uncontrolled PMN infiltration and bystander tissue damage, which are both drivers of pathologic inflammation in mucosal inflammatory disorders.

## Results

### Sialylation-dependent regulation of intestinal PMN TEpM in vitro and in vivo.

**Figure 1:**
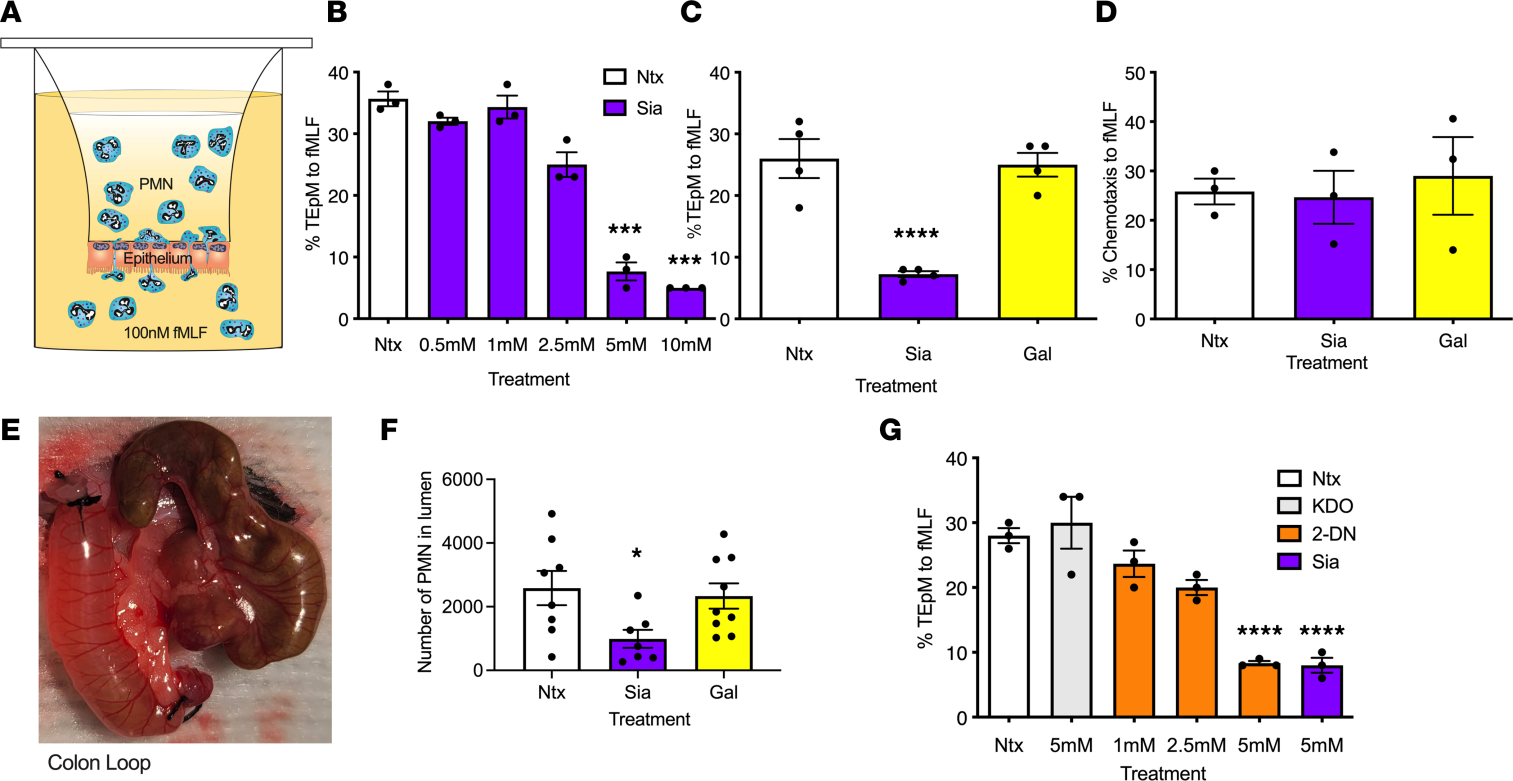
Sialidase inhibition reduces PMN TEpM in vitro and in vivo. (**A**) T84 intestinal epithelial cells were cultured to confluency as inverted monolayers on 3 μm porous polycarbonate filters (Transwell). (**B**) Human PMNs incubated with 0.5 mM to 10 mM Sia were placed in the upper chamber of transwell filters and induced to migrate into the bottom chamber in response to 100 nM fMLF. Migration was quantified by MPO assay. Data are expressed as mean ± SEM and were analyzed by 1-way ANOVA followed by Bonferroni post hoc testing (*n* = 3 independent donors, ***P < 0.001). (**C**) Human PMN were exposed to 5 mM Sia or 5 mM Gal before assessment of TEpM as described above. Data are expressed as mean ± SEM and were analyzed by 1-way ANOVA followed by Bonferroni post hoc testing (*n* = 4 independent donors, ****P < 0.0001). (**D**) Effect of 5 mM Sia or 5 mM Gal on PMN migration across collagen-coated transwells to 100 nM fMLF. Data are expressed as mean ± SEM and were analyzed by 1-way ANOVA followed by Bonferroni post hoc testing (*n* = 3 independent donors). (**E** and **F**) Number of murine PMN recruited into the lumen of proximal colon loops following luminal injection of LTB_4_ ± 5 mM Sia or 5 mM Gal. Data are mean ± SEM and were analyzed by 1-way ANOVA followed by Bonferroni post hoc testing (*n* = 2 independent experiments with 4–5 mice per group, *P < 0.05). (**G**) Human PMNs incubated with 1–5 mM 2-DN, 5 mM KDO, or 5 mM Sia in the upper chamber of transwell filters were induced to migrate into the bottom chamber in response to 100 nM fMLF. Migration was quantified by MPO assay. Data are expressed as mean ± SEM and were analyzed by 1-way ANOVA followed by Bonferroni post hoc testing (*n* = 3 independent donors, ****P < 0.0001).

Given the heavily sialylated nature of PMN surface glycoproteins and previous work showing mobilization of sialidases from intracellular granules to the cell surface of activated PMN (29, 30), studies were performed to determine effects of exogenous free Sia on PMN TEpM. Dose response analyses revealed that exposure of human PMN to 5–10 mM Sia reduced N-formyl-L-methionyl-Leucyl-L-phenylalanine–driven (fMLF-driven) migration across T84 intestinal epithelial cell (IEC) monolayers in the physiologically relevant basolateral to apical direction (Figure 1, A and B). At a concentration of 5 mM, Sia reduced detectable PMN numbers in the apical chamber by ≥ 80% compared with 5 mM control sugar galactose (Gal) (Figure 1C). We next determined effects of Sia on PMN migration in a system with no epithelial cells. For these assays, PMN chemotaxis to 100 nM fMLF across collagen-coated transwells was assessed. In contrast to effects observed during TEpM, exposure to 5 mM Sia had no effect on PMN chemotaxis across collagen-coated transwell filters (Figure 1D). These results suggest that Sia specifically interferes with critical PMN-epithelial adhesion interactions during TEpM.

In vitro experiments were extended to animal studies to see if Sia had similar effects on TEpM in vivo. In these studies, we used a previously established proximal colon loop model that enables quantitative and spatiotemporal studies of leukocyte trafficking across colonic mucosa in response to luminally administered chemoattractants (31). This in vivo surgical model facilitates assessment of PMN at multiple stages of transmigration across intestinal mucosa, including lamina propria and epithelial-associated PMN as well as those that have reached the colonic lumen. Analysis of in vivo murine PMN migration into the proximal colon in response to a solution of luminally applied (leukotriene B_4_) LTB_4_ revealed that coinjection of 5 mM Sia along with LTB_4_ resulted in a ≥ 60% decrease in the number of PMN reaching the intestinal lumen, relative to mice injected with the control sugar Gal (Figure 1, E and F). Given that observed Sia mediated decreases in PMN migration across intestinal mucosa, we hypothesized that addition of free Sia was likely acting as a competitive inhibitor of sialidase activity, thus preventing removal of terminal Sia residues from the cell surface. To confirm that negative effects on TEpM observed with Sia were mediated by sialidase inhibition, PMN were exposed to the pan sialidase inhibitor N-Acetyl-2,3-dehydro-2-deoxyneuraminic acid (2-DN). As can be seen in Figure 1G, incubation of human PMN with 2-DN inhibited TEpM in a dose-dependent fashion, with 5 mM inhibiting migration by ≥ 75% (*P* < 0.0001) compared with 5 mM 2-keto-3-deoxyoctonate ammonium salt (KDO), a molecule with similar charge and structure as 2-DN but without sialidase inhibitory activity (28). To determine if decreases in TEpM observed with sialidase inhibition were the result of PMN aggregation, PMN exposed to 5 mM Sia, 2-DN, or relevant controls were examined by light microscopy. Incubation of PMNs with sialidase inhibitors or control sugars did not result in significant levels of aggregation compared with cells incubated with the known agglutinating agent wheat germ agglutinin (WGA) (Supplemental Figure 1A; supplemental material available online with this article; https://doi.org/10.1172/jci.insight.167151DS1), demonstrating that Sia- or 2-DN–induced inhibition of PMN TEpM in vitro and in vivo is not the result of PMN aggregation. We next determined if inhibition of sialidase activity altered intestinal epithelial barrier function. Exposure of T84 IECs to sialidase inhibitors or controls did not significantly change transepithelial electrical resistance, suggesting that Sia- and 2-DN–mediated decreases in PMN TEpM are not the result of altered epithelial permeability (Supplemental Figure 1B).

### Removal of α2-3 Sia from specific PMN glycoproteins facilitates TEpM.

**Figure 2:**
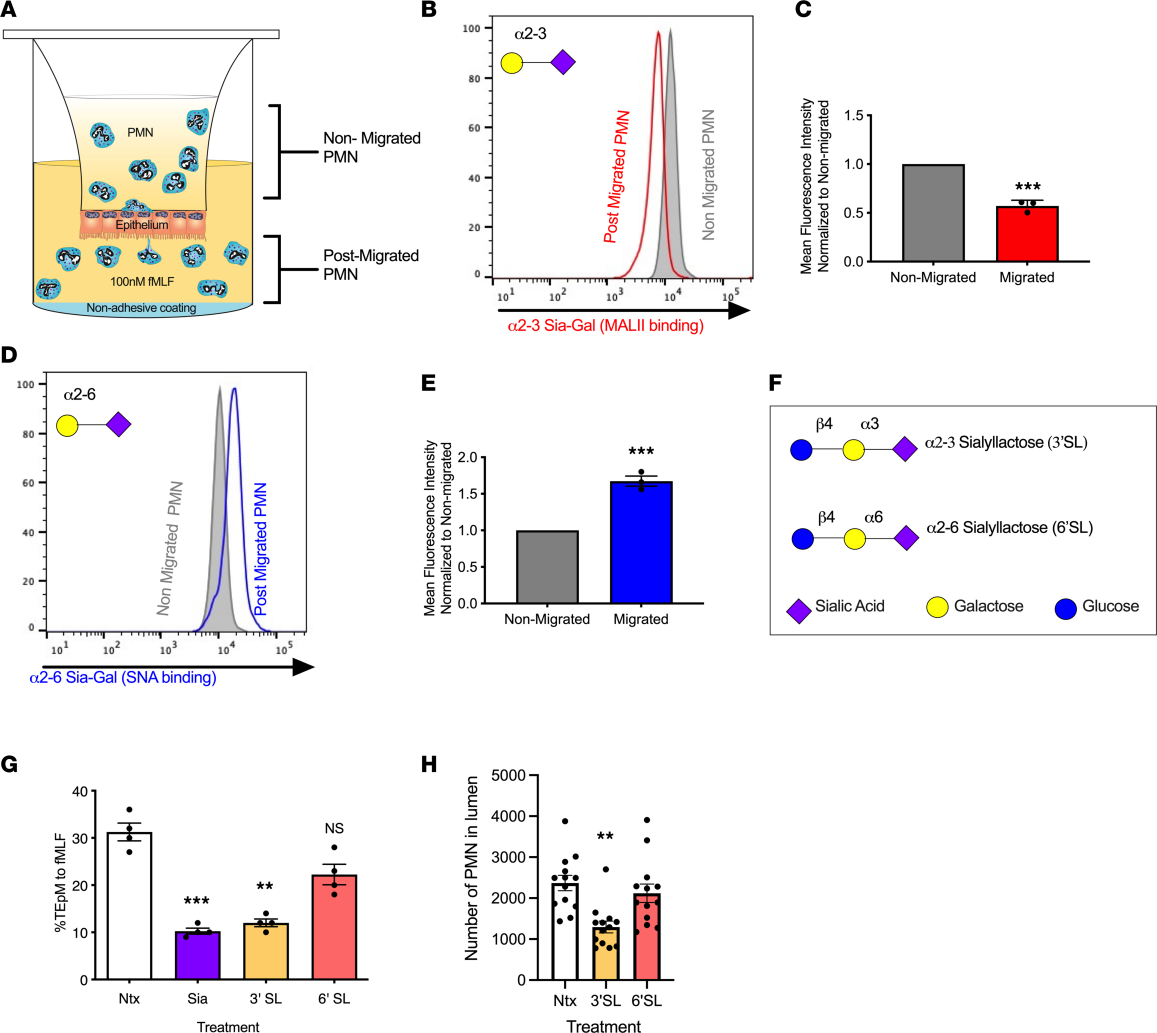
Sialidase-mediated removal of α2-3 Sia is required during PMN TEpM in vitro and in vivo. (**A**–**E**) Levels of surface expression of α2-3– and α2-6–linked Sia were assessed before and after PMN TEpM by flow cytometry using FITC-conjugated MAL II or FITC-conjugated SNA. Data are expressed as mean ± SEM and were analyzed by 1-way ANOVA followed by Bonferroni post hoc testing (*n* = 3 independent PMN donors, ***P < 0.001). (**F** and **G**) Human PMNs incubated with 5 mM 3’SL or 5 mM 6’SL in the upper chamber of transwell filters were induced to migrate into the bottom chamber in response to 100 nM fMLF. Migration was quantified by MPO assay. Data are expressed as mean ± SEM and were analyzed by 1-way ANOVA followed by Bonferroni post hoc testing (*n* = 4 independent donors, **P < 0.01, ***P < 0.001). (**H**) Number of murine PMN recruited into the lumen of proximal colon loops in vivo following luminal injection of LTB_4_ ± 5 mM 3’SL or 5 mM 6’SL. Data are mean ± SEM and were analyzed by 1-way ANOVA followed by Bonferroni post hoc testing (*n* = 3 independent experiments with 3-5 mice per group, **P < 0.01).

Given the reduction in PMN epithelial trafficking mediated by global inhibition of sialidase activity, we analyzed the extent of surface sialylation of human PMN before and after TEpM (Figure 2A). As a common terminating sugar for longer oligosaccharide chains, Sia connects to underlying Gal residues via α2-3 or α2-6 linkages (32, 33). Therefore, we analyzed effects of TEpM on PMN surface expression of α2-3– and α2-6–linked Sia. Interestingly, surface expression of α2-3–linked Sia —as detected by fluorescein isothiocyanate–conjugated (FITC-conjugated) Maackia Amurensis Lectin II (MALII) — decreased by ≥ 40% on postmigrated PMN (Figure 2, B and C). In contrast. a ≥ 60% increase in surface expression of α2-6 Sia — detected by FITC conjugated Sambucus Nigra lectin (SNA) — was observed on PMN that had undergone TEpM (Figure 2, D and E). Data, therefore, demonstrate specific loss of α2-3 sialylation from the surface of PMN that have migrated across IECs. To confirm the requirement for α2-3 sialidase activity during PMN epithelial trafficking, functional effects of either an α2-3 sialidase inhibitor (3’ sialyllactose [3’SL]) or an α2-6–specific sialidase inhibitor (6’SL) were examined (Figure 2F). As can be seen in Figure 2G, inhibition of α2-3 sialidase activity by 3’SL inhibited human PMN TEpM by ≥ 60%, while incubation with 6’SL had no significant effect on PMN epithelial trafficking. Analysis of in vivo migration of murine PMN into the proximal colon in response to a solution of luminally applied LTB_4_ revealed that coinjection of 5 mM 3’SL along with LTB_4_ resulted in a ≥ 55% decrease in the number of PMN reaching the intestinal lumen (Figure 2H). In contrast, coincubation of LTB_4_ with 6’SL had no significant effect on trafficking of murine PMN into the colon. Taken together, results demonstrate the requirement for specific sialidase-mediated removal of α2-3 Sia during PMN trafficking across colonic epithelium in vitro and in vivo.

### CD11b/CD18 is the major PMN glycoprotein decorated with α2-3 Sia, and removal of Sia facilitates CD11b activation.

**Figure 3:**
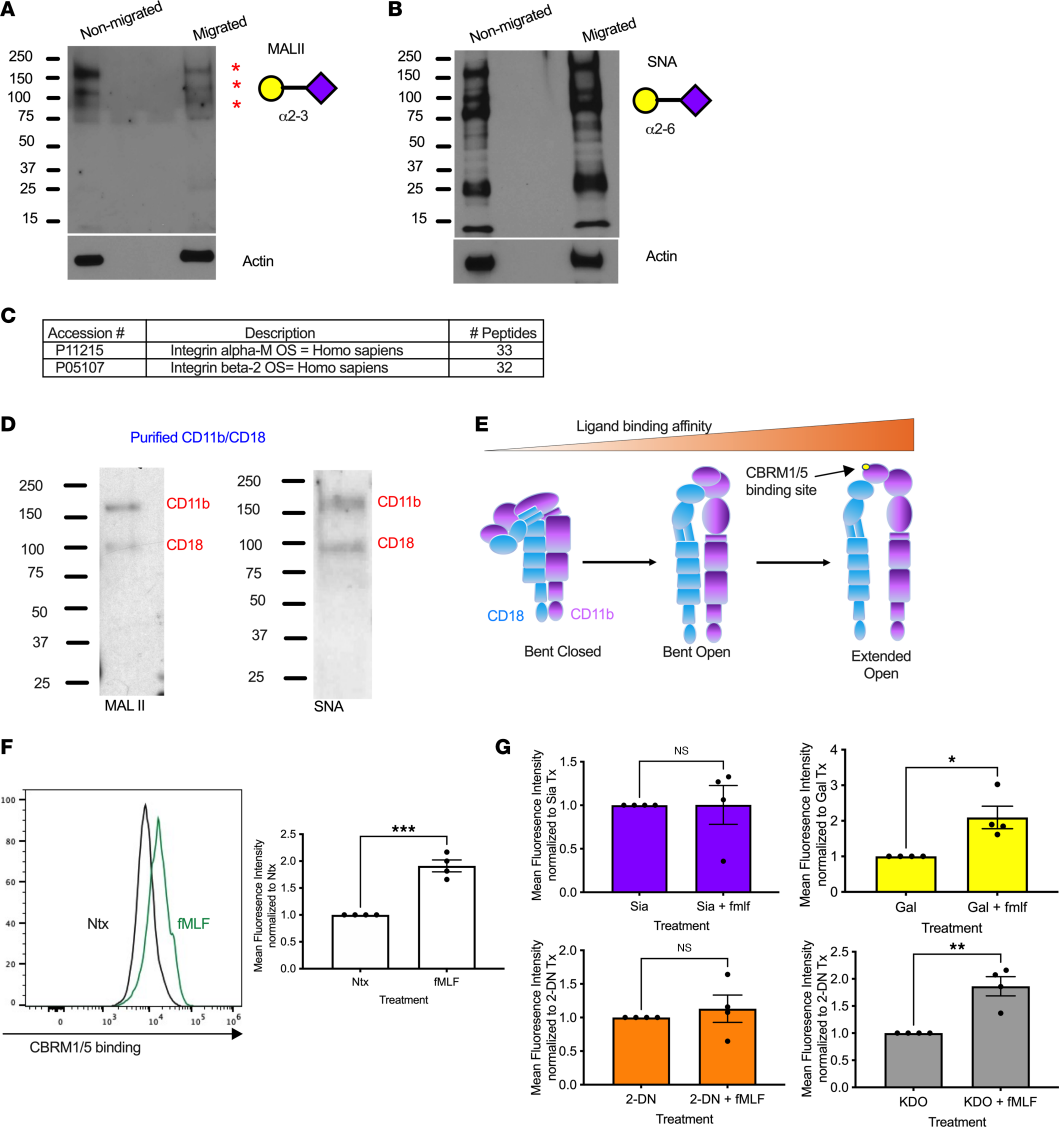
Sialidase inhibition prevents conformational activation of PMN CD11b/CD18. (**A** and **B**) Lysates from PMN before or after TEpM were immunoblotted with biotinylated MALII or SNA. Data shown are representative of PMN from 3 independent donors. (**C**) α2-3 Sia containing glycoproteins were pulled from human PMN lysates by a MALII column and subjected to tryptic digestion and LC-MS/MS analysis. Table shows accession numbers, protein names, and number of tryptic peptides identified. (**D**) In total, 10 μg CD11b/CD18 immunopurified from human PMN was immunoblotted with biotinylated MALII or SNA. (**E**–**G**) Flow cytometry of human PMN following stimulation with 100 nM fMLF ± 5 mM Sia, Gal, 2-DN, or KDO using FITC-conjugated CBRM1/5. Data are expressed as mean ± SEM and were analyzed by 1-way ANOVA followed by Bonferroni post hoc testing (*n* = 4 PMN donors, ***P < 0.001, **P < 0.01, *P < 0.05).

Given the observation that specific removal of α2-3–linked Sia promotes PMN TEpM, experiments were performed to identify human PMN glycoproteins that are preferentially decorated with α2-3– or α2-6–linked Sia by Western blotting with biotinylated MALII or SNA. Three major α2-3 sialylated glycoproteins were identified with molecular weights ranging from 80 to 180 kDa (Figure 3A). Western blotting also confirmed decreased expression of α2-3 Sia by PMN that had migrated across intestinal epithelial monolayers (migrated lane compared with nonmigrated lane, Figure 3A). In contrast to the limited number of α2-3 sialylated glycoproteins observed, immunoblotting with SNA revealed numerous glycoproteins ranging in molecular weight from 15 to 160 kDa that were decorated with α2-6 sialylation. Furthermore, there was no decrease in levels of α2-6 sialylation observed in transmigrated PMN (Figure 3B).

Liquid chromatography tandem mass spectrometry (LC-MS/MS) of glycoproteins isolated by MALII affinity chromatography identified the major carrier of α2-3 sialylation in human PMN lysates to be integrin αM (33 tryptic peptides identified) and β2 integrin (32 tryptic peptides identified) (Figure 3C). These glycoproteins represent both heteromeric subunits of the β2 integrin CD11b/CD18. Western blotting of CD11b/CD18 protein immunopurified from human PMN confirmed α2-3 sialylation and α2-6 sialylation of both CD11b and CD18 subunits (Figure 3D).

It has been previously demonstrated that transition of CD11b/CD18 from a bent conformation into an open extended state facilitates high-affinity integrin binding interactions (Figure 3E) (34). Therefore, we determined whether removal of α2-3 Sia plays a role in CD11b/CD18 conformational activation utilizing flow cytometric analyses and an antibody specific for the active or extended form of CD11b/CD18 (CBRM1/5). As can be seen in Figure 3F, exposure of human PMN to 100 nM fMLF resulted in significantly enhanced CD11b activation. Importantly, exposure of PMN to sialidase inhibitors (5 mM Sia or 5 mM 2-DN) prevented fMLF-mediated increases in CD11b activation (Figure 3G). In contrast, incubation of PMN with controls (Gal and KDO) did not interfere with fMLF-mediated increases in CBRM1/5 binding or CD11b activation (Figure 3G). Taken together, these data suggest that sialidase-mediated removal of α2-3 from CD11b/CD18 plays an important role in CD11b/CD18 conformational activation, thus promoting adhesive interactions during PMN TEpM.

### Sialylation regulates PMN phagocytosis, degranulation, and superoxide release.

**Figure 4:**
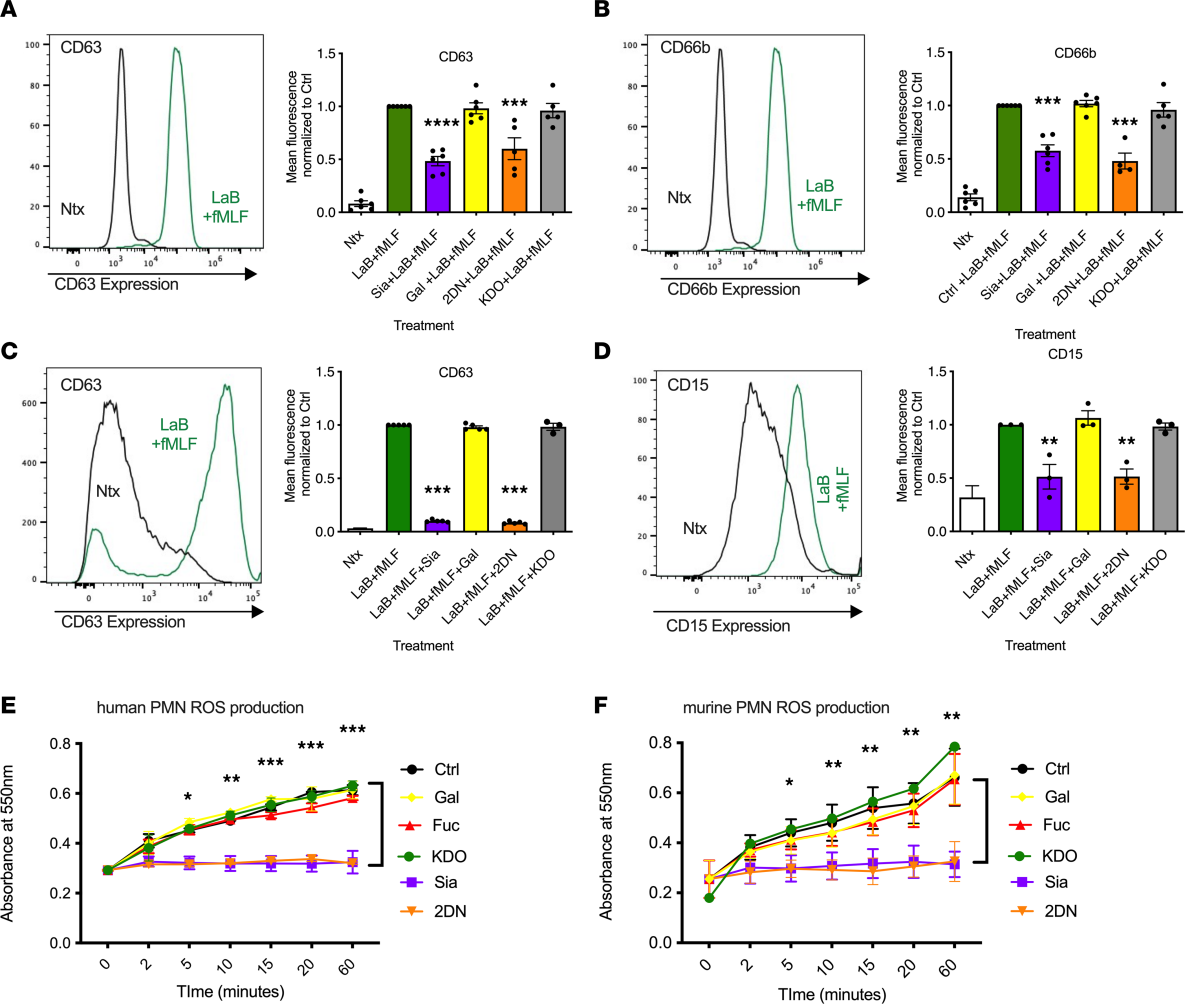
Sialidase inhibition prevents degranulation and ROS release in human and murine PMN. (**A** and **B**) Human PMN were exposed to 5 mM Sia, 5 mM Gal, 5 mM 2-DN, or 5 mM KDO for 30 minutes at 37°C, followed by stimulation with 1.25 μM LaB and 5 μM fMLF to induce degranulation before assessment of surface expression of CD66b and CD63 by flow cytometry. Data shown are fold-change in mean fluorescence intensity (MFI) comparing treatment with sialidase inhibitors against relevant control (Gal or KDO). Data are expressed as mean ± SEM and were analyzed by 1-way ANOVA followed by Bonferroni post hoc testing (*n* = 4–6 PMN donors, ***P < 0.001, ****P < 0.0001). (**C** and **D**) Murine PMN were exposed to 5 mM Sia, 5 mM Gal, 5 mM 2-DN, or 5 mM KDO for 30 minutes at 37°C followed by stimulation with 1.25 μM LaB and 10 μM fMLF to induce degranulation before assessment of surface expression of CD66b and CD63 by flow cytometry. Data shown are fold-change in mean fluorescence intensity (MFI) comparing treatment with sialidase inhibitors against relevant control (Gal or KDO). Data are expressed as mean ± SEM and were analyzed by 1-way ANOVA followed by Bonferroni post hoc testing for PMN isolated from 3–5 mice (**P < 0.01, ***P < 0.001). (**E**) Human PMN incubated with 5 mM Gal, 5 mM Sia, 5 mM 2-DN, or 5 mM KDO were exposed to 100 μM cytochrome C. Reduction of cytochrome C in response to 500 nM fMLF was measured by quantifying changes in absorbance at 550 nm at 2, 5, 10, 15, 20, and 60 minutes. Data are fold change in absorbance relative to time 0, are expressed as mean ± SEM, and were analyzed by 1-way ANOVA followed by Bonferroni post hoc testing (*n* = 3 independent human PMN donors, *P < 0.05, **P < 0.01, ***P < 0.001). (**F**) Murine PMN incubated with 5 mM Gal, 5 mM Sia, 5 mM 2-DN, or 5 mM KDO were exposed to 100 μM cytochrome C. Reduction of cytochrome C in response to 1 μM fMLF was measured by quantifying changes in absorbance at 550 nm at 2, 5, 10, 15, 20, and 60 minutes. Data are fold change in absorbance relative to time 0; data are expressed as mean ± SEM and were analyzed by 1-way ANOVA followed by Bonferroni post hoc testing (*n* = 3 mice, *P < 0.05, **P < 0.01, ***P < 0.001).

Given the important role sialyation plays in PMN TEpM, we examined the effect of inhibiting sialidase activity on other critical CD11b/CD18-mediated PMN inflammatory functions. Effects of sialidase inhibition on PMN degranulation in response to potent stimuli latrunculin B (LaB) and fMLF were evaluated. As expected, and shown in Figure 4, A and B, incubation with 1.25 μM LaB followed by 5 μM fMLF resulted in degranulation, as detected by increased surface expression of markers of primary (CD63) and secondary (CD66b) granules on the surface of human PMN. Importantly, coincubation of PMN with 5 mM Sia or 2-DN significantly reduced LaB and fMLF induced degranulation. In contrast, incubation of PMN with the control sugar Gal or KDO did not reduce degranulation induced by LaB/fMLF treatment. Significant LaB/fMLF-mediated increases in surface expression of markers of primary granules (CD63) and secondary granules (CD15) were also observed in murine PMN (Figure 4, C and D). As was observed for human PMN, coincubation of murine PMN with Sia or 2-DN (Figure 4, C and D) — but not controls (Gal or KDO) — resulted in decreased surface expression of CD63 and CD15 after LaB/fMLF stimulation (Figure 4, C and D). Taken together, these data demonstrate robust sialylation-dependent regulation of human and murine PMN degranulation responses.

PMN oxidative burst responses, while crucial for host defense against invading microbes, are also implicated in PMN-associated tissue damage in numerous inflammatory disorders. As can be seen in Figure 4, E and F, exposure of PMN to the bacterial peptide fMLF resulted in robust superoxide generation (as measured by quantifying reduction of cytochrome C) between 5 and 60 minutes of stimulation. Importantly, coincubation of human or murine PMN with 5 mM Sia or 2-DN significantly decreased fMLF-induced superoxide release at all time points measured between 5 and 60 minutes relative to indicated controls (Figure 4, E and F). In addition to degranulation and reactive oxygen species (ROS) production, phagocytosis is an essential tool in the PMN antimicrobial arsenal. Therefore, we determined effects of sialidase inhibition on PMN phagocytosis. In contrast to inhibitory effects observed for degranulation and superoxide release, flow cytometric analyses demonstrated that exposure of human PMN to 5 mM Sia or 2-DN significantly increased PMN phagocytosis of fluosphere beads relative to relevant controls (Supplemental Figure 2A). A similar increase in PMN phagocytosis of fluorescent beads was observed for murine PMN incubated with 5 mM Sia or 2-DN relative to indicated controls (Supplemental Figure 2B). Given the delayed clearance of PMN in inflamed mucosal tissues under pathologic conditions, we next examined effects of sialidase inhibition on PMN apoptosis. Incubation of human PMN (Supplemental Figure 2C) or murine PMN (Supplemental Figure 2D) with 5 mM Sia had no significant effect on PMN apoptosis levels, as measured by flow cytometry quantification of annexin V^+^ cells. Taken together, these data suggest that sialidase-dependent removal of surface Sia residues is a key driver regulating multiple PMN inflammatory effector functions, including epithelial transmigration, degranulation, and superoxide release.

### Sialylation regulates signaling downstream of β2 integrin in human and murine PMNs.

**Figure 5:**
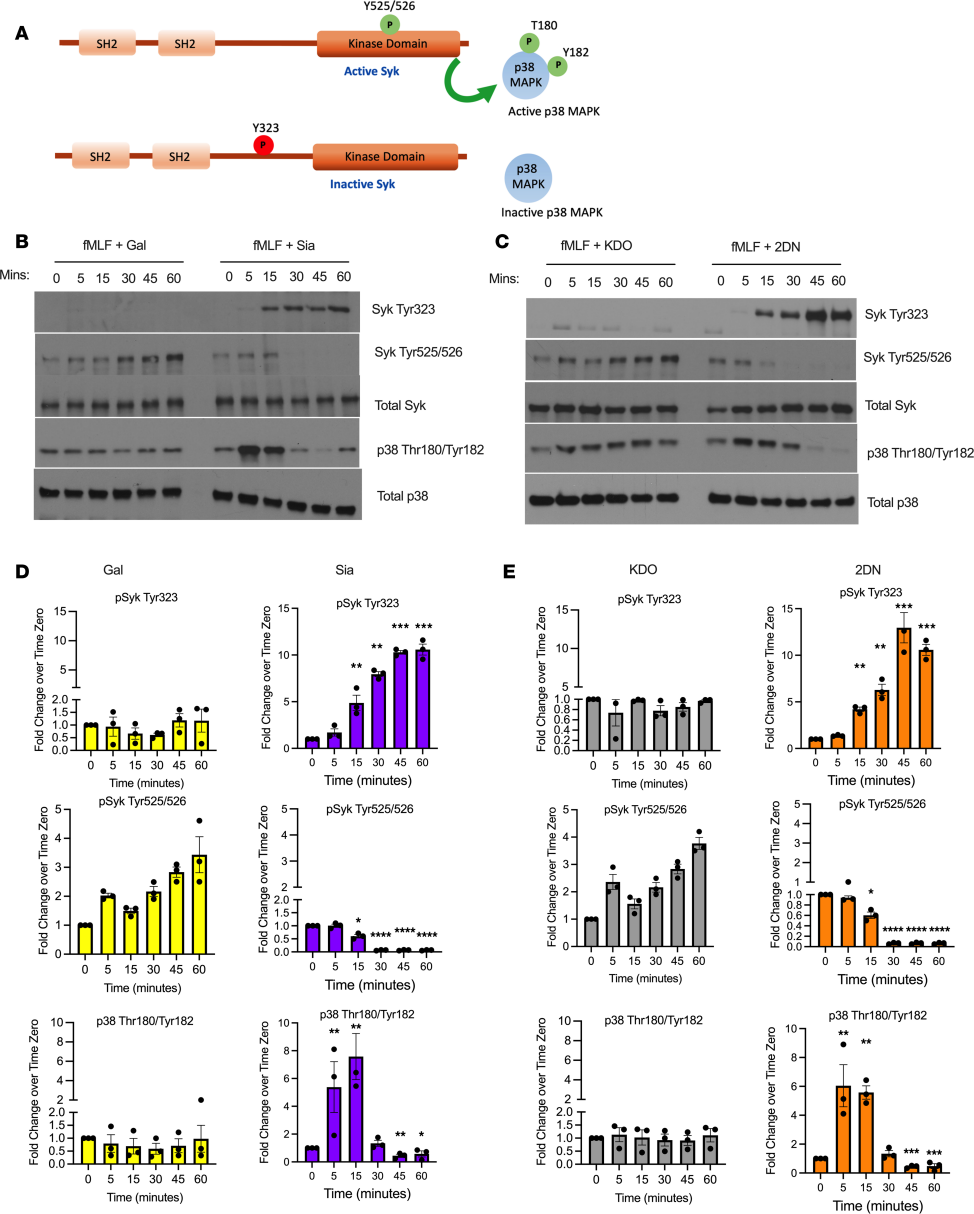
Sialidase inhibition blocks fMLF-mediated Syk and downstream p38 MAPK activation in human PMN. (**A**) Model showing activating and inhibitory phosphorylation sites on CD45 and downstream regulation of p38 MAPK. (**B** and **C**) Lysates from human PMN stimulated over a 60-minute time course with 100 nM fMLF plus 5 mM Gal or 5 mM Sia (**B**), or 5 mM KDO or 5 mM 2-DN (**C**), were immunoblotted for total Syk, Syk^Tyr323^, Syk^Tyr525/526^, total p38 MAPK, or p38^Thr180/Tyr182^. Blots shown are representative of 3 independent PMN donors. (**D** and **E**) Densitometry analyses of phosphorylation of Syk and p38 MAPK following 5, 15, 30, 45, and 60 minutes of stimulation normalized to band intensity at time 0. Data are expressed as mean ± SEM from 3 independent human PMN donors and were analyzed by 1-way ANOVA followed by Bonferroni post hoc testing. *P < 0.05, **P < 0.01, ***P < 0.001, ****P < 0.0001.

**Figure 6:**
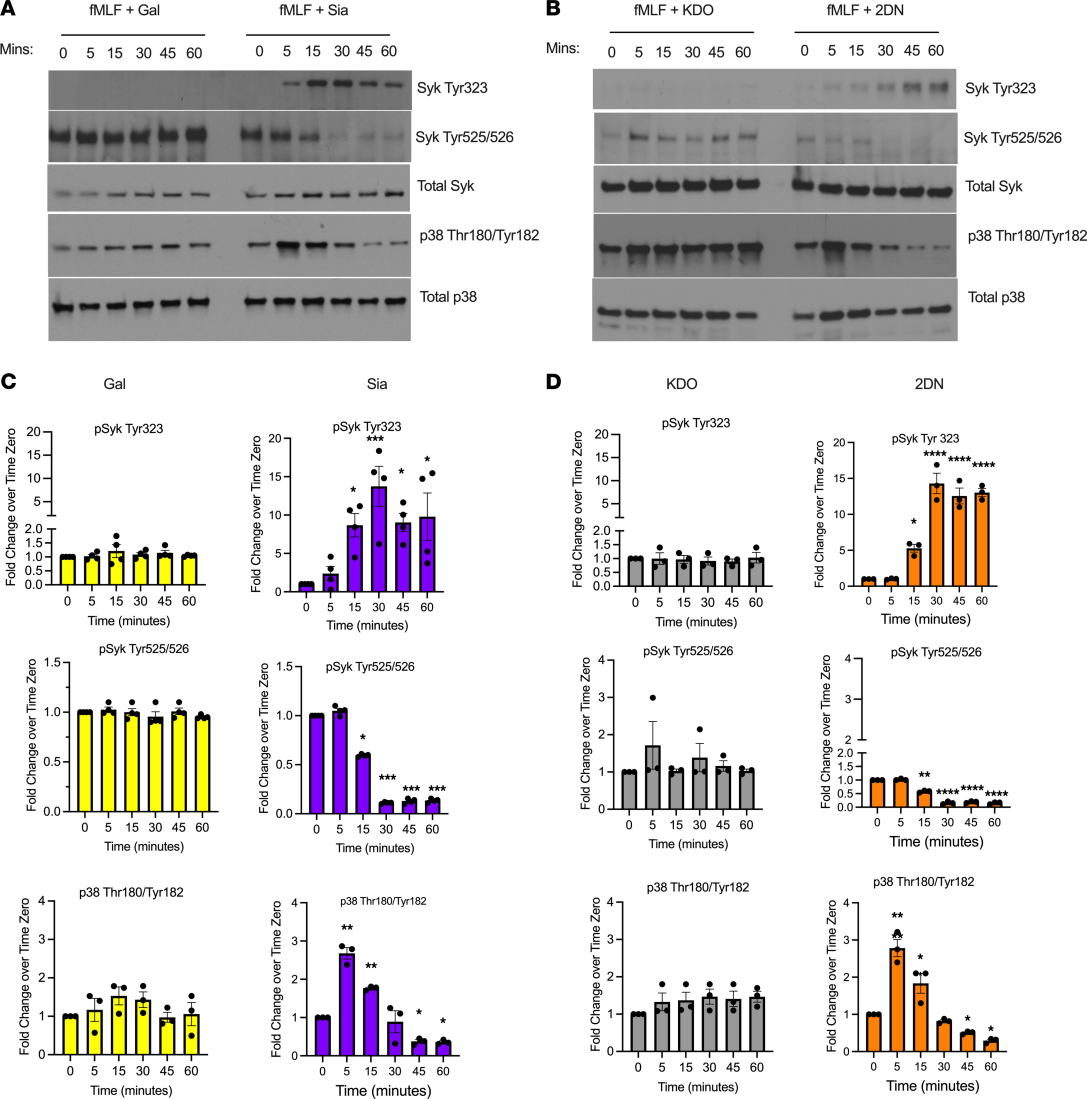
Sialidase inhibition blocks fMLF mediated Syk and downstream p38 MAPK activation in murine PMN. (**A** and **B**) Lysates from murine PMN stimulated over a 60-minute time course with 200 nM fMLF plus 5 mM Gal or 5mM Sia (**A**), or 5 mM KDO or 5 mM 2-DN (**B**), were immunoblotted for total Syk, Syk^Tyr323^, Syk^Tyr525/526^, total p38 ^or^ p38^Thr180/Tyr182^. Blots shown are representative of PMN isolated from 3 to 4 mice. (**C** and **D**) Densitometry analyses of phosphorylation of Syk and p38 MAPK following 5, 15, 30, 45, and 60 minutes of stimulation normalized to band intensity at time 0. Data are expressed as mean ± SEM and were analyzed by 1-way ANOVA followed by Bonferroni post hoc testing (*n* = 3–4 murine PMN donors, *P < 0.05, **P < 0.01, ***P < 0.001, ****P < 0.0001).

**Figure 7:**
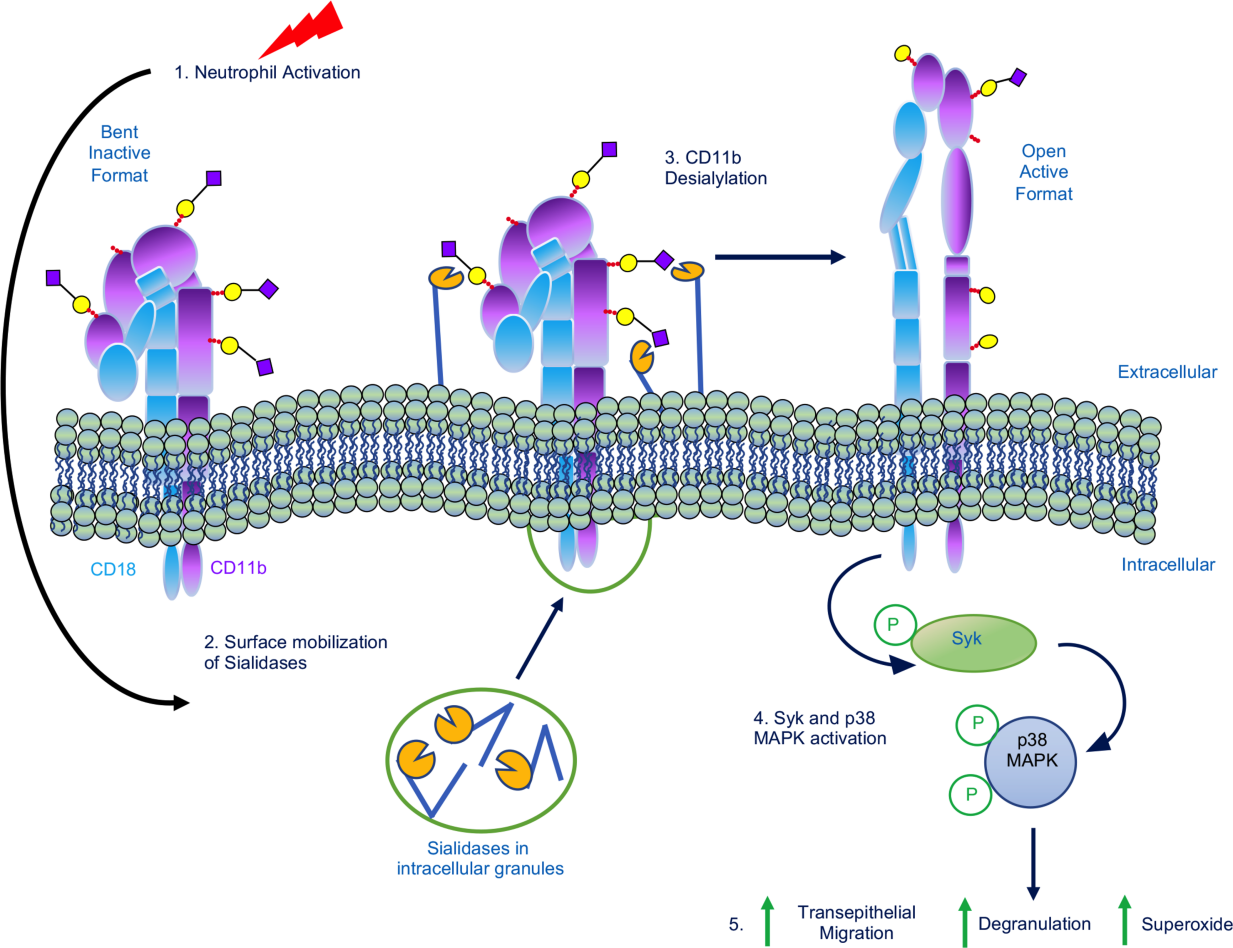
PMN activation model. Model showing how PMN activation results in surface mobilization of intracellular sialidases resulting in desialylation and activation of CD11b/CD18, which signals through Syk and p38 MAPK to drive PMN inflammatory effector functions including TEpM, degranulation and ROS release.

It is well appreciated that activated CD11b/CD18 mediates PMN functions through outside-in signaling via Syk (35). We thus investigated effects of sialyation on Syk signaling in PMN. For these experiments, fMLF-stimulated human and murine PMN were treated with sialidase inhibitors for varying time points, followed by Western blot and probing for changes in Syk activity using antibodies against well-characterized inhibitory (Thr323) and activating (Thr525/526) phosphorylation sites on Syk (Figure 5A) (36, 37). Importantly, coincubation of fMLF-stimulated human PMN with 5 mM Sia or 2-DN resulted in a significant increase in phosphorylation of the inhibitory Syk^Tyr323^ site between 15 and 30 minutes (Figure 5, B, D, and E). In contrast, no phosphorylation of this inhibitory site was observed in PMN stimulated with fMLF plus controls (Gal or KDO). In addition to phosphorylation at Syk^Tyr323^, exposure of PMN to sialidase inhibitors significantly decreased fMLF-mediated phosphorylation of Syk^Tyr525/526^ activation sites between 15 and 60 minutes (Figure 5, B–E). In contrast, consistent increases in Syk^Thr525/526^ phosphorylation were observed in fMLF-stimulated human PMN exposed to Gal or KDO controls with maximal 4-fold increases in Syk activation observed at 60 minutes. It has been previously reported that p38 MAPK signaling is activated downstream of Syk in PMNs (35). We observed that fMLF-mediated activation of p38 MAPK at Thr180 and Tyr182 in human PMN is significantly decreased by sialidase inhibitors (Sia or 2-DN) between 30 and 60 minutes of stimulation (Figure 5, B–E). Consistent with what was observed for human PMN, inhibition of sialidase activity significantly decreased fMLF-mediated activation of Syk (increased Syk^Tyr323^/decreased Syk^Tyr525/526^) in murine PMN at time points between 15 and 30 minutes (Figure 6, A–D). In contrast, robust Syk activation was observed in murine PMN stimulated with fMLF in the presence of controls (Gal or KDO). Sialidase inhibition in murine PMN also resulted in significant decreases in fMLF-induced p38 MAPK activation at time points between 30 and 60 minutes (Figure 6, A–D). These results demonstrate Sia-dependent regulation of Syk-p38 MAPK signaling in activated human and murine PMN. Taken together, these data demonstrate that desialylation activates PMN CD11b/CD18, which signals through Syk and p38 MAPK to upregulate TEpM, superoxide release and degranulation responses (Figure 7).

## Discussion

Dysregulated trafficking of PMN across mucosal surfaces is a hallmark of several inflammatory diseases that are characterized by persistent or intermittent bursts of active inflammation. In the gut, uncontrolled influx of PMNs across intestinal epithelial barriers coupled with indiscriminate release of toxic reactive oxygen metabolites and tissue-degrading proteases results in extensive mucosal and/or transmural injury, including edema, loss of goblet cells, decreased mucus production, crypt injury with erosions, ulceration, and crypt abscess formation that is characteristic of conditions such as ulcerative colitis. Recent studies have demonstrated that specific fucose and GlcNAc glycans can be targeted to downregulate harmful PMN inflammatory effector functions, including TEpM and superoxide generation (26, 27, 38). However, the role of the most abundant terminal glycan (Sia) in regulating PMN inflammatory function remains incompletely understood.

Here, we demonstrate that sialidase-dependent removal of Sia from glycans on the surface of PMNs is required for TEpM into the intestine in vitro and in vivo. This newly identified requirement for sialidase activity during PMN intestinal trafficking is supported by previous studies demonstrating that, upon activation with a variety of stimuli, including PMA, ionomycin, and IL-8, sialidases present within intracellular compartments translocate to the PMN surface, resulting in release of Sia from plasma membrane–bound glycoproteins (29). These data suggest that manipulation of sialidase activity may be a potent tool for reducing PMN influx in chronic inflammatory diseases where recurrent PMN mucosal infiltration is implicated in bystander tissue damage. Indeed, there is a previous report demonstrating that bacterial sialidase inhibitors were effective in reducing the severity of sepsis following cecal ligation in mice (39). In an analogous fashion, inhibitors of viral sialidases such as Relenza (zanamivir) or Tamiflu (oseltamivir) are FDA-approved drugs used to treat and provide prophylaxis against influenza A and B (40, 41) by reducing the efficiency of viral entry into host cells. It has also been reported that mice deficient in 1 of the 4 mammalian sialidases/neuraminidases (42, 43) (Neu3) are protected against a food-poisoning model of colitis involving recurrent infection with *Salmonella typhimurium* (44). This study also found that mice deficient in Neu3 were not protected from DSS-induced colitis, suggesting that other sialidases (which would be inhibited by exogenous Sia or pansialidase inhibitors) play an important role in the pathobiology of intestinal inflammation. The potential relevance of sialidase inhibition to pathological PMN infiltration is further highlighted by work demonstrating that increased sialidase activity in the gut is observed during colitis in vivo and that elevated Sia levels were observed in serum of patients with active Crohn’s disease (45–47).

During posttranslational modification processes, terminal Sia residues are attached to Gal or GalNAc monosaccharides on mammalian glycoproteins via α2-3 or α2-6 bonds created by specific sialyltransferases (48, 49). Previous studies have identified increased surface expression of sialidases as well as transient and rapid decreases in total surface Sia following PMN exposure to nonphysiologic, highly activating agents such as PMA and ionomycin (29, 50). However, selective PMN desialylation has not been examined under physiologically relevant conditions. Here we show specific loss of α2-3 Sia occurring during the physiologically relevant process of TEpM. In keeping with previous work demonstrating that the β2 integrin CD11b/CD18 is a critical mediator of PMN intestinal trafficking in vitro and in vivo (22, 31, 51, 52), we show that CD11b/CD18 is a major carrier of α2-3 sialylation in PMN. In support of desialylation regulating CD11b/CD18 function during PMN activation, it has been reported that PMN stimulation results in close physical approximation of sialidases with CD11b/CD18 on the cell surface (28). Functionally, we show that inhibition of removal of Sia from CD11b/CD18 blocks fMLF-mediated conformational activation of this integrin. While it has been reported that removal of sialyl residues from PMN CD11b/CD18 or ICAM-1 by exogenous sialidases results in enhanced PMN binding to endothelial cells (50), ours is the first report to our knowledge showing that specific loss of α2-3 Sia residues from CD11b/CD18 is required for PMN migration across epithelium. Our data also show that specific inhibition of α2-3 sialidase activity by the milk oligosaccharide 3’SL inhibits PMN intestinal trafficking in vitro and in vivo. The potential utility of naturally occurring milk oligosaccharides as therapeutic agents lacking adverse side effects is exemplified by previous studies demonstrating that 20 g of SL given daily to human subjects was well tolerated, with no negative effects on gastrointestinal function reported (53). In support of this, other work has demonstrated that rats given 3’SL at a dose 2,000 mg/kg body weight experienced no adverse physiological effects (54). Given the biostability of dietary oligosaccharides in the upper GI tract, these regents can reach the distal intestine intact, adding further utility to their potential oral use as antiinflammatory agents (47).

Large-scale intestinal accumulation of PMNs, accompanied by release of tissue-damaging granule contents and production of ROS, are pathological hallmarks of many debilitating conditions, including IBD (2, 6, 7, 55–63). In addition to decreases observed in PMN intestinal epithelial migration, here we report that sialidase inhibition significantly downregulated superoxide release and degranulation responses in human and murine PMN. These data suggest that regulation of surface Sia cleavage has the potential to limit access of PMN to mucosal tissues and to dampen PMN processes implicated in pathological mucosal tissue damage. Consistent with CD11b/CD18 desialylation driving PMN inflammatory function, previous work has demonstrated that CD11b/CD18 modulates PMN superoxide release and degranulation responses (24, 64–66). Glycosylation-mediated regulation of CD11b/CD18 function is further supported by our previous studies showing that lectin targeting of biantennary galactosylated glycans on CD11b results in decreased ROS production in human PMN (27). In addition to CD11b/CD18 glycosylation effecting functional outcomes, previous work has demonstrated that CD11b binding to fucosylated glycans regulates PMN epithelial adhesive interactions in the intestine (52, 67). Interestingly these data highlight that PMN CD11b/CD18 can act both as a ligand for carbohydrate binding proteins and as a lectin-like mediator that can itself engage glycans to effect regulation of cellular functions (68, 69). Interestingly, in contrast to inhibitory effects observed for degranulation and superoxide release, increased levels of PMN phagocytosis were observed downstream of sialidase inhibition. Consistent with effects of sialidase inhibition being secondary to reduced activation of CD11b/CD18, it has previously been reported that PMN from CD11b/CD18-deficient mice do not exhibit decreased levels of phagocytosis of antibody-coated particles (70). Taken together, these studies identify that CD11b/CD18 sialylation is a key mechanism that regulates human and murine PMN mucosal inflammatory effector functions, including TEpM, superoxide release, and degranulation.

Previous reports have identified Syk as a critical downstream signaling mediator for CD11b/CD18. In keeping with this, Syk^–/–^ PMN fail to undergo respiratory burst, degranulation, or spreading in response to proinflammatory stimuli (35, 71, 72). Here we show that, in addition to downregulating CD11b/CD18 activation, sialidase inhibition reduced fMLF-mediated activation of Syk signaling in human and murine PMN. A key downstream signaling pathway element of Syk in PMN is p38 MAPK (71). Importantly, our results show that deactivation of Syk observed following exposure of human or murine PMN to sialidase inhibitors coincided with decreased activation of p38 MAPK. Taken together, these data suggest that, upon PMN stimulation, enhanced surface sialidase activity serves to remove Sia from CD11b/CD18, and this removal facilitates conformational integrin activation and subsequent outside-in signaling mediated by Syk and p38 MAPK that drives PMN inflammatory effector functions, including migration, degranulation, and superoxide release. Inhibition of CD11b/CD18 desialylation may represent a promising therapeutic strategy for reducing dysregulated PMN influx and associated mucosal tissue damage in chronic inflammatory disorders, including COPD and IBD.

## Methods

### Antibodies, lectins, and reagents.

SNA, MALII, and protein free blocking solution were purchased from Vector Labs. Polymorphprep was purchased from Axis-Shield. In total, 1 μm FITC-conjugated FluoSpheres (505/515) were purchased from Molecular Probes. N-formyl-L-methionyl-Leucyl-L-phenylalanine (fMLF), polyhydroxyethylmethacrylate, LaB, Cytochrome c from bovine heart, Sia, Gal, 2-DN, and KDO were purchased from Sigma-Aldrich. Both 3’SL and 6’SL were purchased from Cayman Chemical. FITC-conjugated CD11b activation specific monoclonal antibody (CBRM1/5) was purchased from Thermo Fisher Scientific (catalog 14-0113-81). Signaling antibodies for immunoblotting were purchased from Cell Signaling Technologies (total Syk, 2712; Syk Tyr323, 2715; Syk Tyr525/526, 2710; total p38 MAPK, 9212; and p38 MAPK Thr180/Tyr182, catalog 4511). Human TruStain FcX (Fc receptor blocking solution) was purchased from BioLegend. Mouse anti–human FITC–conjugated anti-CD63 (catalog 550759) and anti-CD66b (catalog 561927) mAbs were purchased from BD Bioscience Rat anti–mouse PE-conjugated CD63 (catalog 12063182) and anti-CD15 (catalog MA1-022) mAbs were purchased from Thermo Fisher Scientific.

### Cell culture and PMN isolation.

Cultures of T84 IECs were grown as described previously (26, 38, 73). Human PMN were isolated from whole blood obtained from healthy male and female volunteers, with approval from the University of Michigan IRB on human subjects, using a previously described Polymorphprep density gradient centrifugation technique (73, 74). Isolated PMN were 98% pure and > 95% viable and were used for all described assays within 2 hours of blood draw. Murine neutrophils were isolated from bone marrow extracted from the femur and tibias of male and female C57BL/6 mice using EasySep kits from Stem Cell Technologies as previously described (75).

### PMN immunoblotting and CD11b/CD18 purification.

Human and murine PMN cell lysates were boiled in sample buffer under reducing conditions and then subjected to SDS-PAGE, followed by overnight transfer to PVDF. Binding of primary antibodies or biotinylated lectins was detected with HRP-conjugated secondary antibodies or HRP-conjugated streptavidin, respectively. Data represent averages for 3–5 independent PMN donors. Functionally active CD11b/CD18 was immunopurified from human PMNs by LM2/1 immunoaffinity chromatography as described previously (27).

### PMN transmigration and chemotaxis assay.

T84 IECs were grown on collagen-coated, permeable 0.33 cm^2^ polycarbonate filters (3 μm pore size), as inverted monolayers and effects of 5 mM indicated sugars or inhibitors on human PMN TEpM to 100 nM fMLF accessed in the physiologically relevant basolateral to apical direction by MPO quantification (26, 38, 73, 74). For chemotaxis assays, human PMNs were incubated with 5 mM indicated sugars/inhibitors before migration across collagen-coated, permeable 0.33 cm^2^ polycarbonate filters to 100 mM fMLF was assessed by measurement of MPO as described above.

### PMN TEpM into the colon in vivo.

Colon loop experiments were performed with male and female C57BL/6 mice aged 8–12 weeks that were maintained under standard conditions with 12-hour light/12-hour dark cycles and had ad libitum access to food and water. PMN TEpM into the proximal colon was assessed as described previously (31, 76). Briefly, mice were pretreated with proinflammatory cytokines before a 2 cm loop of fully vascularized proximal colon was injected with 1 nM LTB_4_ and 5 mM Sia, 5 mM 3’SL, 5 mM 6’SL, or relevant controls before migration of PMNs into the colon lumen was quantified by flow cytometry.

### Flow cytometry and PMN phagocytosis assay.

For analysis of changes in surface sialylation during PMN TEpM, nonmigrated PMN and PMNs that had migrated across T84 IEC monolayers into wells coated with antiadhesive poly(2-hydroxyethyl methacrylate) were collected. PMN were blocked in 3% BSA before incubation with 10 mg/mL FITC-labeled SNA or MALII. For assessment of CD11b/CD18 activation, human PMN with or without stimulation with fMLF were blocked with 3% BSA containing Human TruStain FcX (Fc Receptor Blocking Solution; BioLegend) before incubation with FITC-conjugated CBRM1/5. Flow cytometric analyses were performed using a NovoCyte Flow cytometer (ACEA bioscience). For phagocytosis assays, PMN were incubated with 5 mM indicated sugars or 2-DN before addition of FITC conjugated FluoSpheres at a ratio of 1:100 (PMN/FluoSpheres) in the presence of 10 nM fMLF. Uptake of FluoSpheres by PMNs was assessed by measurement of fluorescence by flow cytometry, as described above.

### PMN degranulation assay.

Murine and human PMNs were isolated as described above and incubated with 5 mM indicated sugars or inhibitors for 30 minutes at 37°C. As a positive control for degranulation, PMNs were exposed to 1.25 μM LaB for 5 minutes, followed by stimulation with either 5 μM fMLF (human PMN) or 10 μM fMLF (murine PMN). After indicated incubations, PMN were washed in human or murine TruStain Fc receptor blocking solution (FcX) and incubated with FITC or phycoerythrin-conjugated (PE-conjugated) mAbs against CD63 (catalog 550759), CD66b (561927), or CD15 (catalog 561072) (as indicators of primary and specific granules) before data acquisition using a novocyte Flow cytometer (ACEA Bioscience).

### PMN superoxide generation assay.

For measurement of oxidative burst, 0.25 ***×*** 10^6^ human PMNs or 0.5 ***×*** 10^6^ murine PMNs were exposed to 5 mM indicated sugar, inhibitor, or control in HBSS^+^ (plus indicates the presence of calcium and magnesium in the buffer) containing 100 mM cytochrome C as described previously (26, 27). Reduction of cytochrome C following addition of 500 nM fMLF (human PMN) or 1 μM fMLF (murine PMN) was assessed by measuring changes in absorbance at 550 nm at indicated time points using a Cytation 5 imaging reader (BioTek).

### MALII column pulldown and protein identification by LC-MS/MS.

For enrichment of sialylated glycoproteins, lysates from human PMN were passed over a MALII lectin spin column, and proteins containing α2-3 Sia were eluted according to manufacturer’s instructions (Qproteome Sialic Glycoprotein Kit). For in-solution digestion, proteins were reduced, alkylated, and incubated overnight with trypsin, followed by desalting using a SepPak C18 cartridge. Samples were dried via vacufuge, and resulting peptides were resolved on a nano-capillary reverse phase column (Acclaim PepMap C18). Eluent was directly introduced into an Orbitrap Fusion Tribrid mass spectrometer (Thermo Fisher Scientific) using an EasySpray source. MS1 scans were acquired at 120,000 resolution, and data-dependent collision–induced dissociation MS/MS spectra were acquired using the Top-speed method following each MS1 scan (77). Individual α2-3 sialylated glycoproteins were identified by searching the MS/MS data against Homo Sapien Uniprot reviewed entries using Proteome Discoverer (v2.1, Thermo Fisher Scientific). Search parameters included MS1 tolerance of 10 ppm and fragment tolerance of 0.2 Da. FDR was determined using percolator, and proteins/peptides with a FDR of 1% were retained for further analysis.

### Statistics.

Statistical analyses were performed using Prism software (GraphPad Software Inc.). A 2-tailed Student’s *t* test was used in case of parametric parameters. Differences were evaluated by 1-way ANOVA followed by Bonferroni post-hoc test. *P* < 0.05 was considered statistically significant. Data are presented as means ± SEM. All results show data from at least 3 independent experiments.

### Study approvals.

All experimental procedures involving mice were performed in accordance with NIH guidelines and protocols approved by the University Committee on Use and Care of Animals at University of Michigan. All experiments using human blood were conducted in accordance with NIH guidelines approved by the IRB of the University of Michigan Medical School. For human blood draw experiments, written consent was obtained prior to participation.

## Author contributions

JCB designed the study, performed data collection, and performed data analysis/interpretation. MK, DF, and VA performed experiments for the study. JCB wrote the manuscript. RDC provided assistance with interpretation of glycan binding data. AN and CAP provided assistance in writing the manuscript.

## Supplementary Material

Supplemental data

## Version 1 01/31/2023

In-Press Preview

## Version 2 03/08/2023

Electronic publication
