# Supplementary material for: Sialylation regulates neutrophil transepithelial migration, CD11b/CD18 activation, and intestinal mucosal inflammatory function
Source: JCI Insight. 2023 Mar 8;8(5):e167151. doi: 10.1172/jci.insight.167151 (PMC10077474; doi:10.1172/jci.insight.167151)
Supplement: Supplemental data [file jciinsight-8-167151-s124.pdf]

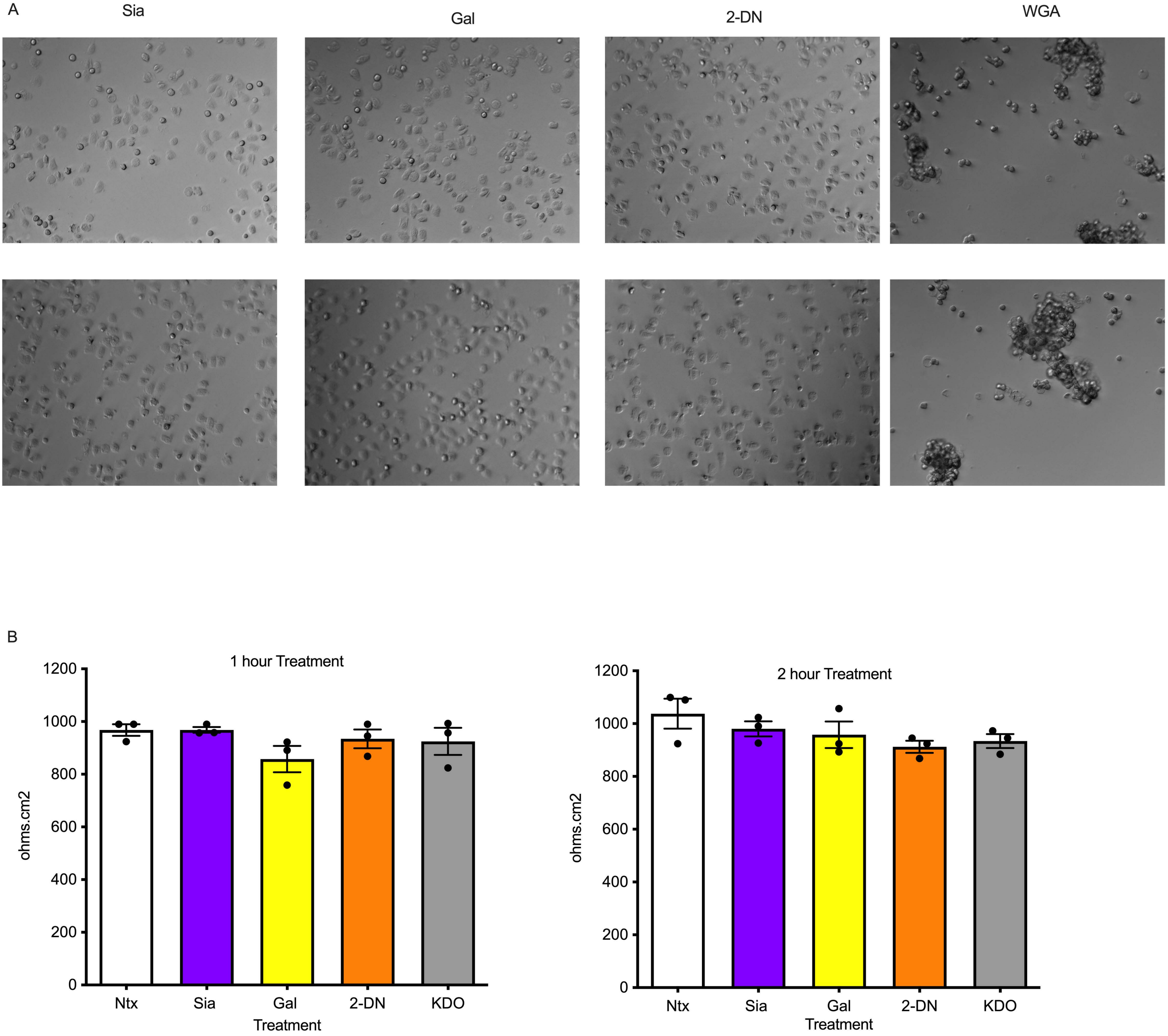

Supplemental Figure 1. A) Human PMN were incbubated with 10μg/ml Sia, Gal, 2-DN or WGA for 60 minutes at 37°C before assessment of aggregation by light microscopy. Data are representative of PMN isolated from n=3 independent donors. B) T84 IEC monolayers on 0.33cm2 transwell filters were incubated with 10μg/ml Sia, Gal. 2-DN or KDO for 1 hr or 2 hr at 37°C and electrical resistance readings captured by an Epithelial Volt/Ohm (TEER) Meter. Data shown are ohms.cm2 and are average for 3 independent experiments with 6 transwell filters per treatment group.

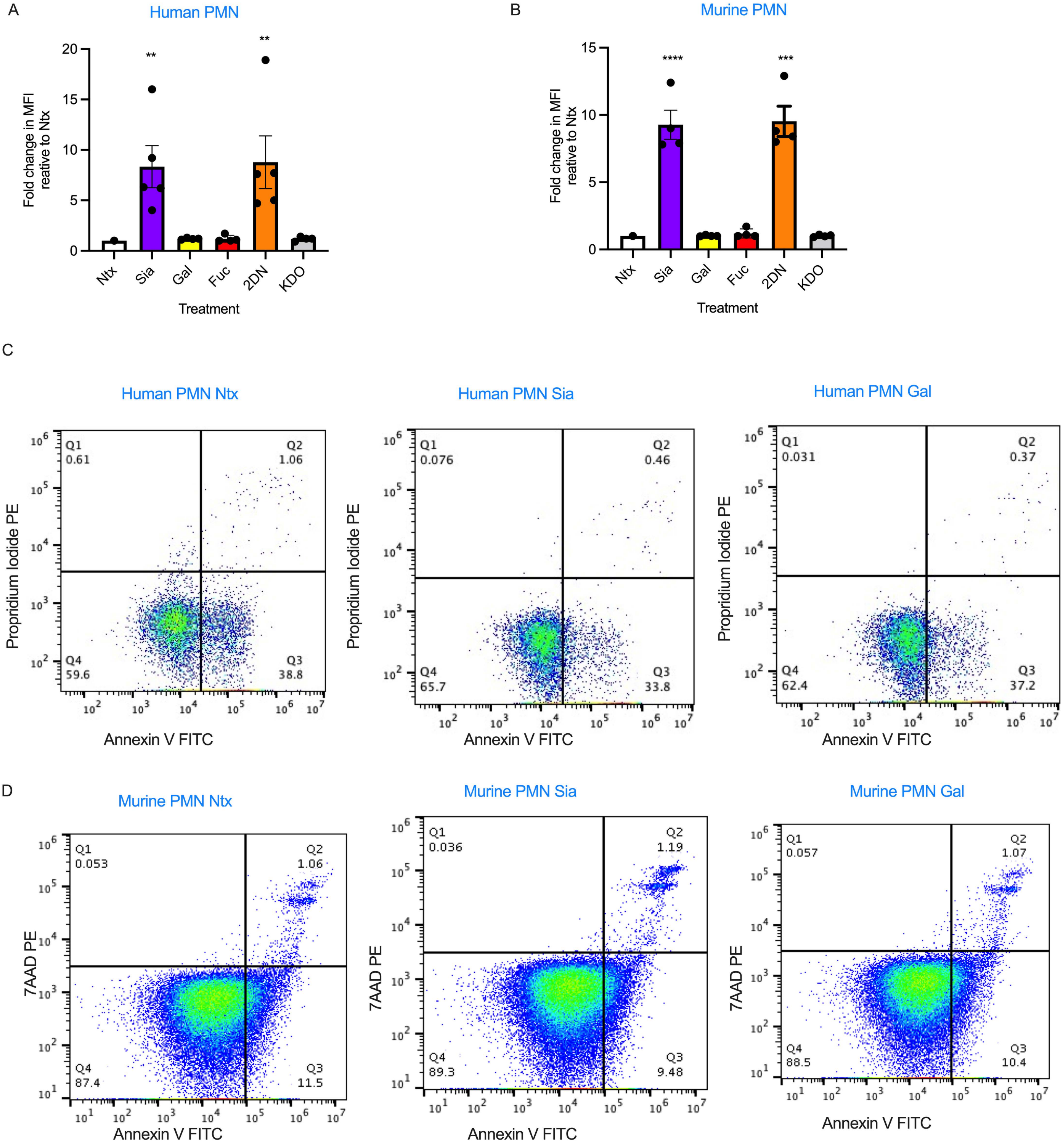

Supplemental Figure 2. Human PMN (A) or Murine PMN (B) were incubated with 5mM Sia or Gal for 60 min at 37°C before fluorescent microsphere phagocytosis/uptake was quantified by measuring changes in fluorescence by flow cytometry. Data are mean fluorescence intensity normalized to non-treated PMN and are expressed as mean ± SEM. n = 3 independent donors, **P <0.01, *** P<0.001, **** P<0.0001. (B) For apoptosis assays, human or murine PMN were incubated with 5mM Sia or Gal for 60 minutes at 37°C before assessment of surface expression of Annexin V and 7AAD by flow cytometry. Cells negative for Annexin IV and 7AAD were considered non-apoptotic. Representative flow plots show percentage apoptotic PMN under the defined conditions.
